# Survival of Bordetella bronchiseptica in Acanthamoeba castellanii

**DOI:** 10.1128/spectrum.00487-23

**Published:** 2023-03-27

**Authors:** Dendi Krisna Nugraha, Takashi Nishida, Yuki Tamaki, Yukihiro Hiramatsu, Hiroyuki Yamaguchi, Yasuhiko Horiguchi

**Affiliations:** a Department of Molecular Bacteriology, Research Institute for Microbial Diseases, Osaka University, Suita, Osaka, Japan; b Department of Medical Laboratory Science, Faculty of Health Sciences, Hokkaido University, Sapporo, Hokkaido, Japan; c Center for Infectious Disease Education and Research, Osaka University, Suita, Osaka, Japan; University of North Dakota

**Keywords:** *Bordetella bronchiseptica*, *Acanthamoeba*, survival, Bvg phase, extrahost, coculture, adhesin

## Abstract

The respiratory pathogenic bacterium Bordetella bronchiseptica can persistently survive in terrestrial and aquatic environments, providing a source of infection. However, the environmental lifestyle of the bacterium is poorly understood. In this study, expecting repeated encounters of the bacteria with environmental protists, we explored the interaction between B. bronchiseptica and a representative environmental amoeba, Acanthamoeba castellanii, and found that the bacteria resisted amoeba digestion and entered contractile vacuoles (CVs), which are intracellular compartments involved in osmoregulation, to escape amoeba cells. In prolonged coculture, A. castellanii supported the proliferation of B. bronchiseptica. The avirulent Bvg^−^ phase, but not the virulent Bvg^+^ phase, of the bacteria was advantageous for survival in the amoebae. We further demonstrate that two Bvg^+^ phase-specific virulence factors, filamentous hemagglutinin and fimbriae, were targeted for predation by A. castellanii. These results are evidence that the BvgAS two-component system, the master regulator for Bvg phase conversion, plays an indispensable role in the survival of B. bronchiseptica in amoebae.

**IMPORTANCE** The pathogenic bacterium Bordetella bronchiseptica, which causes respiratory diseases in various mammals, exhibits distinct Bvg^+^ and Bvg^−^ phenotypes. The former represents the virulent phase, in which the bacteria express a set of virulence factors, while the role of the latter in the bacterial life cycle remains to be understood. In this study, we demonstrate that B. bronchiseptica in the Bvg^–^ phase, but not the Bvg^+^ phase, survives and proliferates in coculture with Acanthamoeba castellanii, an environmental amoeba. Two Bvg^+^ phase-specific virulence factors, filamentous hemagglutinin and fimbriae, were targeted by A. castellanii predation. B. bronchiseptica turns into the Bvg^–^ phase at temperatures in which the bacteria normally encounter these amoebae. These findings demonstrate that the Bvg^–^ phase of B. bronchiseptica is advantageous for survival outside mammalian hosts and that the bacteria can utilize protists as transient hosts in natural environments.

## INTRODUCTION

Bordetella bronchiseptica is a problematic pathogen in veterinary medicine, causing respiratory diseases in various domestic animals, such as swine, dogs, cats, and rabbits, and occasionally immunocompromised humans (1). This organism is categorized as one of the “classical” *Bordetella* species, together with the closely related bacterium Bordetella pertussis, the causative agent of whooping cough (2). The pathogenicity of these *Bordetella* species is regulated by a master regulator, the BvgAS two-component system (3). At the optimal growth temperature of 37°C, the BvgAS system is active and promotes the expression of various virulence factors. Conversely, this system is inactivated at temperatures below 26°C or in the presence of modulating agents, such as nicotinic acid or magnesium sulfate, thus repressing the expression of virulence factors. The former bacterial state is called the Bvg^+^ phase (virulent phenotype), and the latter is the Bvg^−^ phase (avirulent phenotype). It is known that the Bvg^+^ phase is essential for the bacteria to infect mammalian hosts. The Bvg^−^ phase is considered necessary for environmental survival outside mammalian hosts (4–7), but how it is advantageous in extrahost environments is unknown.

The survival of classical *Bordetella* species in natural environments has often been shown with B. bronchiseptica: B. bronchiseptica proliferates at 10°C and 37°C in nonnutrient lake water and phosphate-buffered saline (PBS), respectively, and remains viable for up to 12 weeks (8, 9). Another study demonstrated that it grew for 72 h in soil extracts at 25°C (2). 16S rRNA-based nucleotide sequences retrieved from the National Center for Biotechnology Information (NCBI) database revealed that *Bordetella* species are frequently found in various soil and water samples (2). Phylogenetic analyses also suggested that the ancestor of *Bordetella* species, to which B. bronchiseptica is considered mostly related in classical *Bordetella*, had inhabited soil and water. In addition, one seminal study showed that B. bronchiseptica survives within the amoeba Dictyostelium discoideum for several generations and disseminates from the amoeba after extended periods (10). That study also demonstrated that the bacteria retain the ability to infect mice even after repeated passages through the amoeba. These results indicate that B. bronchiseptica survives in natural environments along with soil amoebae. It is also conceivable that soil organisms, such as *Dictyostelium*, which inhabit the natural environment and may recurrently encounter bacteria, serve as niches for B. bronchiseptica. However, how B. bronchiseptica avoids predation by the amoeba and proliferates there requires further study.

In the present study, we examined the fate of B. bronchiseptica upon its encounter with Acanthamoeba castellanii, which is more ubiquitous in terrestrial and aquatic habitats and has a simpler life cycle than D. discoideum. Microscopic analyses demonstrated that B. bronchiseptica was internalized by amoebae and transferred into contractile vacuoles (CVs), from which the bacteria directly escaped to the extracellular milieu. The survival ability of B. bronchiseptica in the amoeba was dependent on the Bvg phase: the Bvg^+^ phase-locked mutant was preferentially transported to the intracellular digestion pathway of the amoeba compared to the Bvg^−^ phase-locked mutant. By screening bacterial genes that are related to survival ability, we found that two bacterial adhesion molecules, filamentous hemagglutinin (FHA) and fimbriae (FIM), are targeted for predation by the amoeba. These results indicate that the Bvg^−^ phase, in which the expression of the target molecules for the amoeba is shut down, is advantageous for survival in amoebae.

## RESULTS

### B. bronchiseptica proliferates with the aid of A. castellanii.

**FIG 1:**
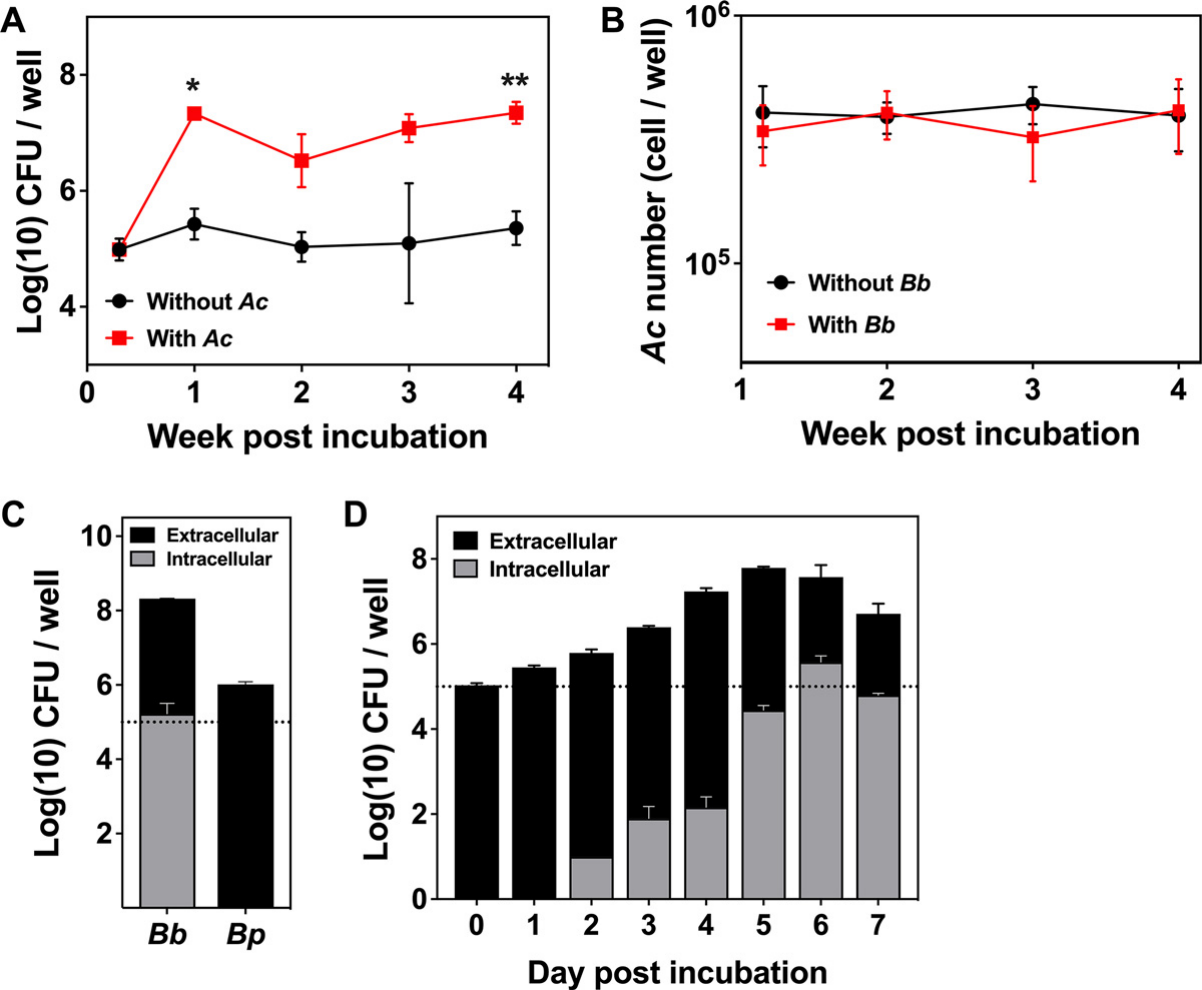
The presence of A. castellanii stimulated B. bronchiseptica growth in HG. (A) The survival of B. bronchiseptica in the presence (red) or absence (black) of A. castellanii (*Ac*) in HG. B. bronchiseptica was incubated with A. castellanii at an MOI of 1. The total CFU of the bacteria was enumerated at the indicated time points. (B) The total number of A. castellanii trophozoites after incubation in the presence (red) or absence (black) of B. bronchiseptica (*Bb*). A. castellanii was incubated with B. bronchiseptica at an MOI of 100, and the total number of amoeba cells at the indicated time points was counted after 0.2% trypan blue staining. (C) B. bronchiseptica (*Bb*), but not B. pertussis (*Bp*), survived intracellularly in A. castellanii after coculture. The bacteria were incubated with A. castellanii at an MOI of 1 in HG. Total (black plus gray) and intracellular (gray) CFU were enumerated on day 7 of the coculture. (D) Intracellular B. bronchiseptica was increased during coculture with A. castellanii in HG for 7 days. B. bronchiseptica was incubated with A. castellanii at an MOI of 1. Total (black plus gray) and intracellular (gray) bacteria were enumerated at each time point. Values represent the mean ± SD (*n* = 3). Statistical differences were analyzed by two-way analysis of variance (ANOVA) with Šídák’s multiple-comparison test (A). *, *P* < 0.05; **, *P* < 0.01. The dotted line indicates the initial inoculation size.

B. bronchiseptica survived in HG (50 mM HEPES buffer, pH 7.4, containing 5 mM glucose), as mentioned in Materials and Methods (also shown in Fig. 1A). It also proliferated within the first 7 days in coculture with A. castellanii. The number of A. castellanii trophozoites in HG was unchanged regardless of the presence of B. bronchiseptica after 4 weeks of coculture (Fig. 1B), indicating that the bacteria did not affect A. castellanii viability. After 7-day coculture, B. bronchiseptica was recovered from inside the amoeba (Fig. 1C). B. bronchiseptica within A. castellanii was first detected 2 days postinfection and gradually increased to reach a peak on day 6 (~10^5^ CFU/well) (Fig. 1D). In contrast, B. pertussis was not recovered from A. castellanii, although the total number of B. pertussis bacteria was slightly increased after coculture with A. castellanii for 7 days, implying that B. pertussis does not reside in but survives outside the amoeba (Fig. 1C).

### Intracellular localization of B. bronchiseptica in A. castellanii.

**FIG 2:**
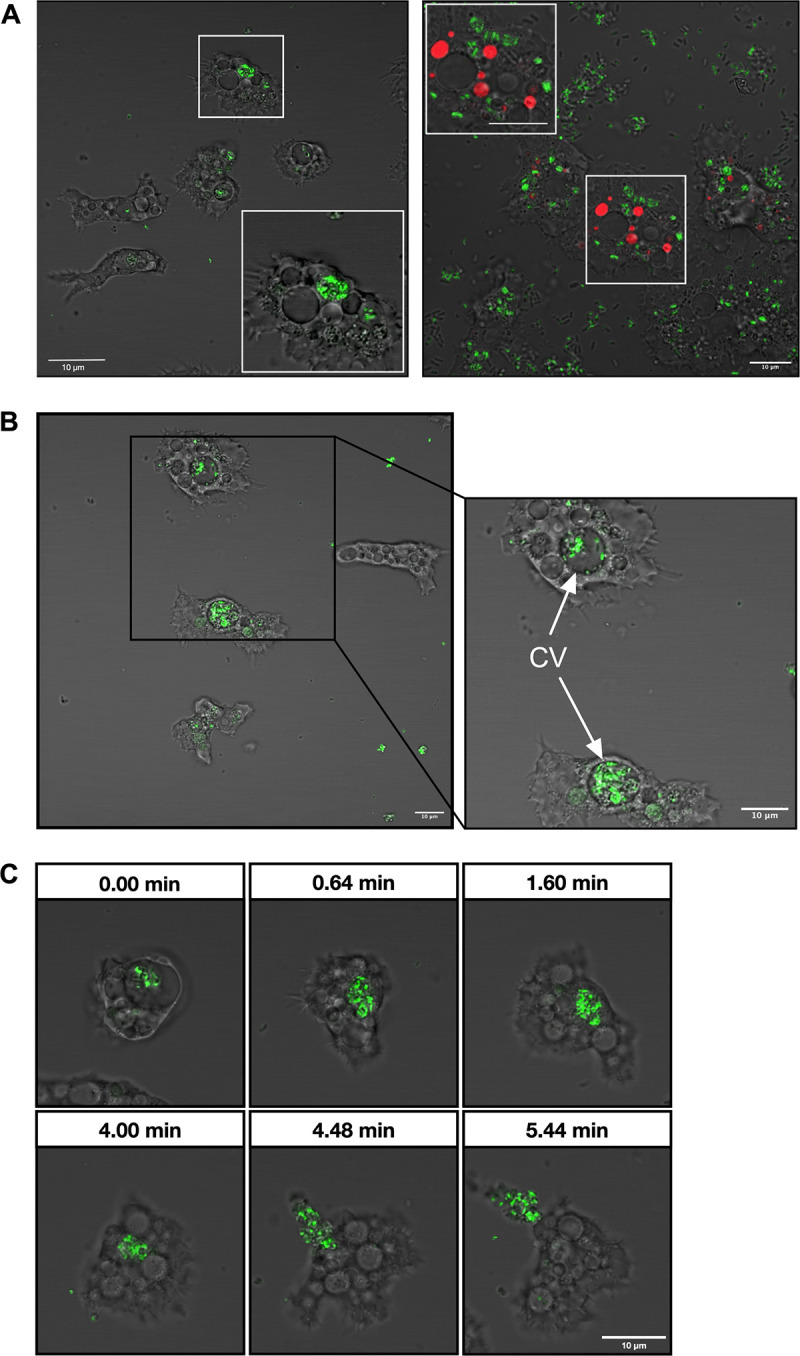
B. bronchiseptica escaped A. castellanii digestion by localizing in CVs. (A) GFP-expressing B. bronchiseptica was accumulated within specific vacuoles (left). These vacuoles were discriminated from those containing red dextran (right). B. bronchiseptica was incubated with A. castellanii at an MOI of 3,000 in HG containing 40 μg/mL dextran and subjected to microscopy at 6 h postinfection. (B) GFP-expressing B. bronchiseptica was localized within large and clear CVs (white arrows). B. bronchiseptica was incubated with A. castellanii at an MOI of 1,000 in HG at 20°C for 7 days. (C) Time-lapse images showing intracellular B. bronchiseptica escaped from a CV expelling its contents probably through the water expulsion process. The relative time points are indicated. Bar, 10 μm.

To trace the fate of B. bronchiseptica in A. castellanii, we cocultured green fluorescent protein (GFP)-expressing bacteria with amoebae. After 6 h of coculture, the majority of the intracellular bacteria were localized within vacuoles not labeled with Alexa Fluor-labeled, high-molecular-weight dextran (Fig. 2A and see Fig. S1 in the supplemental material), a marker for food vacuoles (FVs) in the phagoendosomal pathway of A. castellanii (11). After 7 days, bacteria were found within CVs, which were readily discriminated from other vacuoles by their large and clear appearance under a phase-contrast microscope (Fig. 2B). The CV is a cellular compartment that regulates the intracellular osmolarity of amoeba cells by periodically collecting excess cytosolic water and expelling it to the extracellular milieu (12, 13). Because CVs are independent of the phagoendosomal pathway of the amoebae, bacteria likely evade amoeba digestion by localizing to CVs. Time-lapse imaging revealed that bacteria localized within CVs were eventually expelled to the extracellular milieu along with the CV content (Fig. 2C and Movie S1). These results indicate that internalized B. bronchiseptica bacteria survive A. castellanii digestion by utilizing CVs.

### B. bronchiseptica in the Bvg^−^ phase is advantageous for survival in A. castellanii.

**FIG 3:**
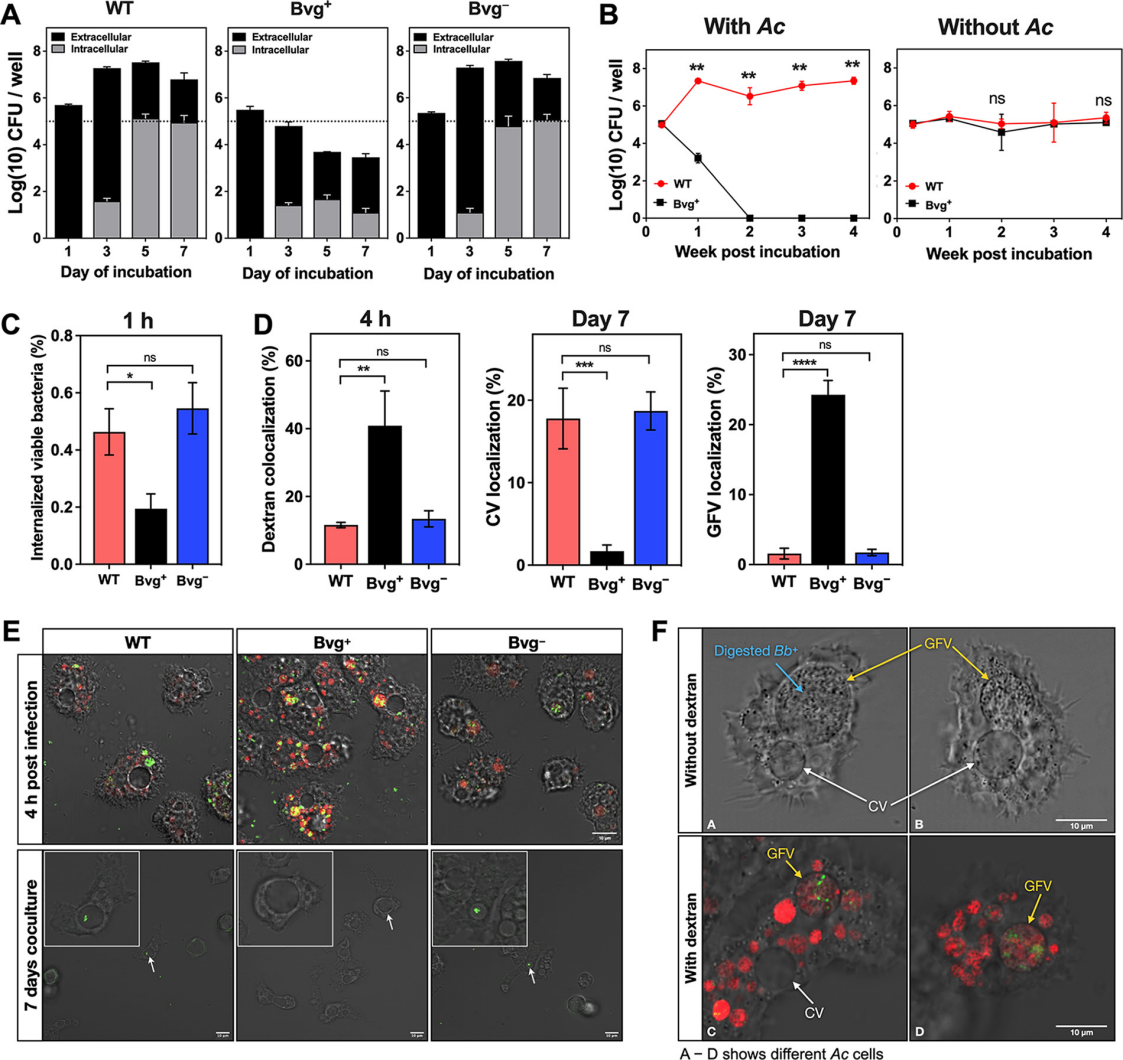
The Bvg^−^ phase is advantageous for B. bronchiseptica to survive in coculture with A. castellanii. (A) The total (black plus gray) and intracellular (gray) number of B. bronchiseptica WT (left) and Bvg^+^ phase-locked (center) and Bvg^−^ phase-locked (right) mutants at each time point recovered from coculture with A. castellanii with an initial MOI of 1. The dotted line indicates the initial inoculation size. (B) The Bvg^+^ phase-locked mutant was eliminated in coculture with A. castellanii for prolonged periods. B. bronchiseptica WT or Bvg^+^ phase-locked mutant was incubated for 4 weeks in the presence of A. castellanii at an initial MOI of 1. The total bacteria at each time point were enumerated. (C) The Bvg^−^ phase was advantageous for B. bronchiseptica survival in A. castellanii. B. bronchiseptica WT (pink) and Bvg^+^ phase-locked (black) and Bvg^−^ phase-locked (blue) mutants were incubated for 1 h in the presence of A. castellanii at an MOI of 100, and intracellular bacteria were enumerated. (D) The Bvg^+^ phase-locked mutant was transferred to the amoeba digestion pathway. Amoebae cultivated in a 35-mm dish were infected with GFP-expressing B. bronchiseptica WT (pink) and Bvg^+^ phase-locked (black) and Bvg^−^ phase-locked (blue) mutants in HG at an MOI of 1,000 (for 7 days of coculture) or 3,000 (for 4 h of infection) with or without 40 μg/mL Alexa Fluor-labeled dextran at 20°C. At the indicated incubation periods, the specimens were examined under a confocal microscope, and several fields of view (~400 amoeba cells) were analyzed for each group. The frequency of positive localization of GFP-expressing B. bronchiseptica strains in dextran-filled FVs (left), CVs (center), and GFVs (right) is expressed as a percentage relative to the total number of amoeba cells. (E) Representative images of GFP-expressing B. bronchiseptica strains internalized in A. castellanii. A. castellanii was infected with B. bronchiseptica strains at an MOI of 1,000 in HG supplemented with 40 μg/mL dextran. Colocalization of GFP-expressing B. bronchiseptica strains and dextran within FVs (orange) was observed at 4 h postinfection (top). GFP-expressing B. bronchiseptica strains were incubated with A. castellanii at an MOI of 1 in HG at 20°C, and the images were taken at day 7 of the incubation (bottom). CVs in the amoebae are indicated by white arrows (bottom) and magnified (inset). (F) Debris of dead Bvg^+^ phase-locked mutants was accumulated within GFVs. The GFP-expressing Bvg^+^ phase-locked mutant was incubated with A. castellanii (*Ac*) at an MOI of 1,000 for 7 days. Dextran was added to the culture 4 h before the microscopy. Dextran-filled GFVs and CVs are indicated by yellow and white arrows, respectively. Debris of dead bacteria found in GFVs is indicated by blue arrow. Bars represent the mean ± SD (*n* = 3 for panels A, B, and C). Statistical differences were analyzed by two-way analysis of variance with Šídák’s multiple-comparison test (B) or one-way ANOVA with Tukey’s multiple-comparison test (C and D). *, *P* < 0.05; **, *P* < 0.01; ***, *P* < 0.001; ****, *P* < 0.0001; ns, not significant.

We next investigated whether the survival of B. bronchiseptica in A. castellanii is dependent on the Bvg phase. Bvg^+^ phase- and Bvg^−^ phase-locked mutants of B. bronchiseptica, which constitutively express the Bvg^+^ and Bvg^−^ phenotypes (4), respectively, showed no effects on the viability of A. castellanii trophozoites compared with wild type (WT) cocultured with the amoebae (Fig. S2). These results suggest that major virulence factors expressed in the Bvg^+^ phase, such as FHA, FIM, adenylate cyclase toxin (CyaA), pertactin, dermonecrotic toxin (DNT), and the type III secretion system, were not toxic to A. castellanii. Furthermore, we found that the Bvg^−^ phase-locked mutant, but not the Bvg^+^ phase-locked mutant, proliferated and survived after 7 days of coculture with A. castellanii, similar to WT (Fig. 3A). The Bvg^+^ phase-locked mutant, which survived in HG, was eliminated within 2 weeks in the presence of A. castellanii (Fig. 3B). After as little as 1 h of coculture, less recovery of the Bvg^+^ phase-locked mutant was achieved from the amoebae than that of the B. bronchiseptica WT or Bvg^−^ phase-locked mutant, as judged by gentamicin protection assay (GPA) (Fig. 3C). Microscopic analyses revealed that the Bvg^+^ phase-locked mutant was noticeably more colocalized with Alexa Fluor-labeled dextran than was the WT or the Bvg^−^ phase-locked mutant after 4 h, suggesting that the Bvg^+^ phase-locked mutant was preferentially transported to the amoeba digestion pathway (Fig. 3D, left, and Fig. 3E, top). The Bvg^−^ phase-locked mutant, but not the Bvg^+^ phase-locked mutant, was found in CVs, similar to WT (Fig. 3D, center, and Fig. 3E, bottom). When the GFP-expressing Bvg^+^ phase-locked mutant was applied along with mCherry-expressing B. bronchiseptica WT at a 1:1 ratio to coculture with A. castellanii, only mCherry-expressing B. bronchiseptica WT was found in CVs after 7 days of incubation (Fig. S3). Furthermore, the GFP-expressing Bvg^+^ phase-locked mutant was hardly observed in the extracellular medium after 7 days (Fig. S3). These results are consistent with those shown in Fig. 3B: the Bvg^+^ phase-locked mutant is likely phagocytized and digested by A. castellanii. Indeed, after 7 days of coculture, dead cells of the Bvg^+^ phase-locked mutant (observed as granule-like substances) were accumulated within dextran-labeled large vacuoles (Fig. 3F), which were previously defined as giant food vacuoles (GFVs), specific compartments involved in the amoeba phagoendosomal pathway (14). We also confirmed that these GFVs were distinct from CVs, as dextran was accumulated only within GFVs. Colocalization of bacteria in GFVs was hardly observed in coculture with WT or the Bvg^−^ phase-locked mutant (Fig. 3D, right). From these results, we concluded that the Bvg^−^ phase is advantageous for B. bronchiseptica to avoid predation by A. castellanii.

### A. castellanii targets FHA and FIM expressed by B. bronchiseptica for predation.

**FIG 4:**
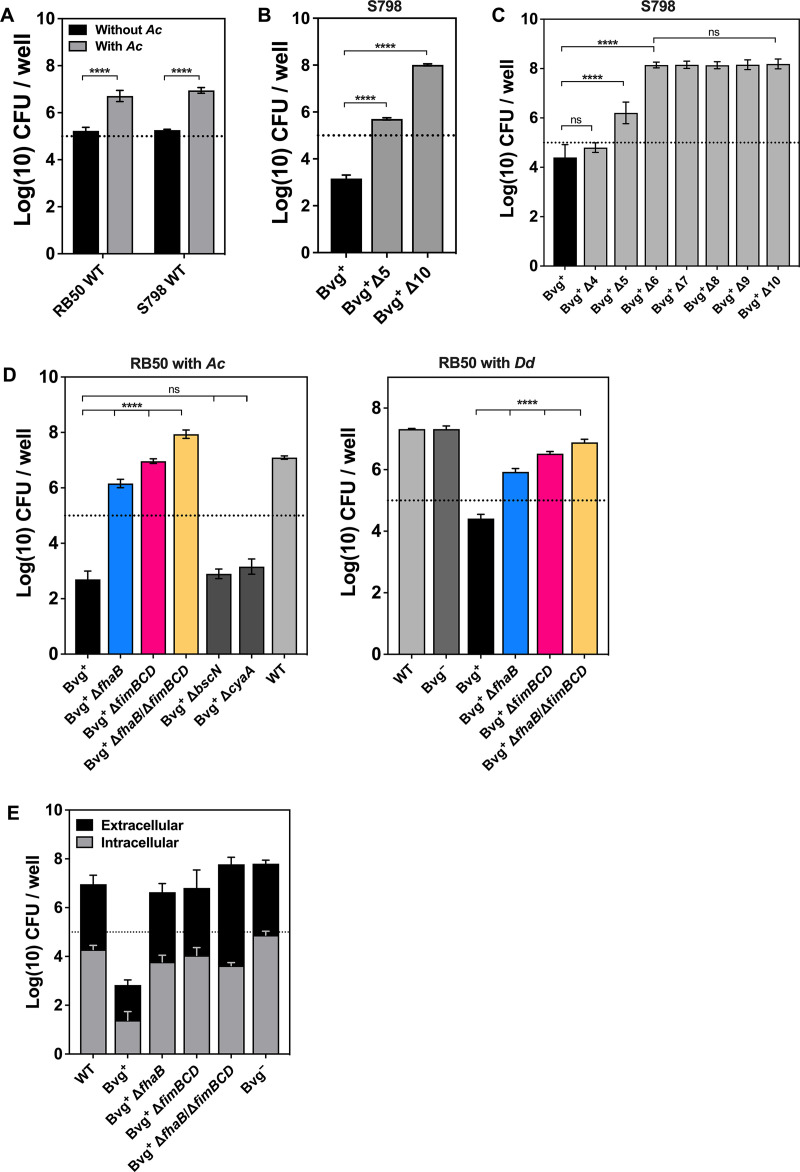
Survival of B. bronchiseptica deficient in Bvg^+^ phase-specific genes in coculture with A. castellanii. B. bronchiseptica strains and mutants were incubated with amoebae at an MOI of 1. After a 7-day coculture, the total CFU of the bacteria was enumerated. (A) Comparison between the number of B. bronchiseptica S798 and RB50 strain bacteria recovered after the culture with (gray) or without (black) amoebae. (B and C) Survival of B. bronchiseptica S798 polymutants after coculture with amoebae. (D and E) Survival of B. bronchiseptica RB50 mutants in coculture with A. castellanii (D, left, and E) or D. discoideum (*Dd*) (D, right). Intracellular bacteria were independently recovered and enumerated (E, gray). Values represent the mean ± SD (*n* = 3). Statistical differences were analyzed by two-way analysis of variance with Šídák’s multiple-comparison test (A) or one-way ANOVA with Tukey’s multiple-comparison test (B, C, and D). ****, *P* < 0.0001; ns, not significant. The dotted line indicates the initial inoculation size.

We next explored why the Bvg^+^ phase-locked mutant, but not the Bvg^−^ phase-locked mutant, served as prey for the amoebae. There are two possible explanations: factors specifically expressed in the Bvg^−^ phase protect the bacteria from predation, or Bvg^+^ phase-specific factors are recognized by the amoebae for digestion. In this study, we examined the latter possibility because Bvg^−^ phase-specific factors are currently elusive compared to Bvg^+^ phase-specific factors, which are widely known to include virulence factors. To this end, we used polymutant strains based on Bvg^+^ phase-locked mutants of the S798 strain kept in the laboratory (15). These mutants were deficient in multiple Bvg^+^ phase-specific virulence factors (here referred to as B. bronchiseptica polymutants) (Table 1). The S798 strain survived in coculture with A. castellanii (Fig. 4A), similar to the RB50 strain used in the experiments described elsewhere in this paper. We first examined the survival ability of Bvg^+^ phase-locked Δ5 and Δ10 strains in the coculture assay and recovered them from the coculture at levels approximately 2- and 4-log-fold higher than that of the isogenic Bvg^+^ phase-locked mutant, respectively (Fig. 4B). Next, a series of poly-mutant strains, Δ4 to Δ10, were examined. After coculture, Bvg^+^ phase-locked Δ6, Δ7, Δ8, Δ9, and Δ10 were highly recovered (~10^8^ CFU/well) compared to Δ4, which was recovered at a level similar to the isogenic Bvg^+^ phase-locked mutant. Bvg^+^ phase-locked Δ5 was recovered intermediately between the isogenic Bvg^+^ phase-locked mutant and Δ6 (Fig. 4C). Bvg^+^ phase-locked Δ5 was generated by the deletion of *fhaB* encoding filamentous hemagglutinin preprotein FhaB (16) in Δ4, and Δ6 was generated from Δ5 by the additional deletion of *fimBCD*, which encodes chaperone FimB, outer membrane usher protein precursor FimC, and minor fimbrial adhesin FimD (17) (Table 1). Therefore, we considered whether *fhaB*- and *fimBCD*-encoded factors are targeted by A. castellanii. Consistent results were obtained in RB50-based mutants: the Bvg^+^ phase-locked RB50 mutant had lower survival in coculture with A. castellanii than RB50 WT, and the deletion of *fhaB* and/or *fimBCD* resulted in a better survival of the Bvg^+^ phase-locked mutant in A. castellanii, in which the total number of Bvg^+^ phase-locked Δ*fimBCD* bacteria was significantly higher than that of Bvg^+^ phase-locked Δ*fhaB* bacteria (Fig. 4D, left, and Fig. S4). In addition, the total CFU of Bvg^+^ phase-locked Δ*bscN* and Δ*cyaA* strains, which served as negative controls, was comparable to that of the isogenic Bvg^+^ phase-locked mutant (Fig. 4D, left). The intracellular Bvg^+^ phase-locked Δ*fhaB* and Δ*fimBCD* strains were recovered from A. castellanii to a similar extent as the WT and the Bvg^−^ phase-locked mutant (Fig. 4E). These results indicate that FHA and FIM encoded by *fhaB* and *fimBCD*, respectively, are targeted for predation by A. castellanii. Similar results were obtained in coculture with D. discoideum (Fig. 4D, right), indicating that involvement of FHA and FIM in predation is not peculiar to A. castellanii.

**TABLE 1:** List of targeted virulence genes for screening

Mutant	Genotype	Gene group	Gene locus	Gene function (accession no. AP014582.1)
Δ1	Δ*bcsN*	T3SS^*b*^	BBS798_1587	Type III secretion system ATPase
Δ2	Δ1 Δ*dnt*	Toxin	BBS798_3771	Dermonecrotic toxin
Δ3	Δ2 Δ*prn*	Autotransporter	BBS798_1328	Pertactin precursor
Δ4	Δ3 Δ*cyaA*	Toxin	BBS798_0319	Bifunctional hemolysin-adenylate cyclase
Δ5	Δ4 Δ*fhaB*	Adhesin	BBS798_2822	Filamentous hemagglutinin
Δ6	Δ5 Δ*fimBCD*	Adhesin	BBS798_2820	Chaperone protein (*fimB*)
			BBS798_2819	Outer membrane usher protein precursor (*fimC*)
			BBS798_2818	Minor fimbrial adhesin (*fimD*)
Δ7	Δ6 Δ*vag8*	Autotransporter	BBS798_1765	Virulence associated gene 8
Δ8	Δ7 Δ*fhaS*	Adhesin	BBS798_2151	Filamentous hemagglutinin-like protein
Δ9	Δ8 Δ*fhaL*	Adhesin	BBS798_1837	Filamentous hemagglutinin-like protein
Δ10	Δ9 Δ*brtA*	Adhesin	BBS798_1147	Adhesin^*a*^

a Homolog to *Bordetella* RTX-family adhesin (BrtA) of B. bronchiseptica RB50 (accession number NC_002927.3) (42).

b T3SS, type III secretion system.

### Bvg^+^ phase-locked B. bronchiseptica lacking *fhaB* or *fimBCD* was transferred to A. castellanii CVs, similar to WT.

**FIG 5:**
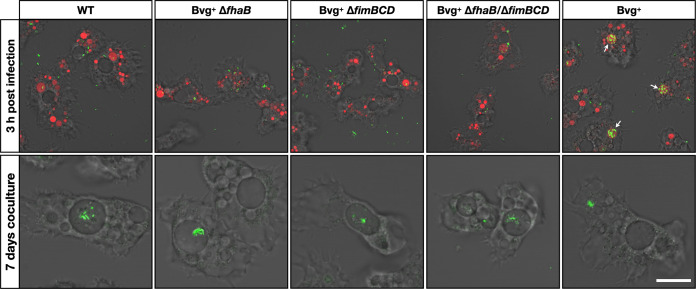
Bvg^+^ phase-locked B. bronchiseptica lacking *fhaB* and/or *fimBCD* was transferred to CVs similarly to WT. (Top) Evasion by Bvg^+^ phase-locked B. bronchiseptica lacking *fhaB* and/or *fimBCD* of FVs. (Bottom) Localization of Bvg^+^ phase-locked B. bronchiseptica lacking *fhaB* and/or *fimBCD* to CVs. GFP-expressing Bvg^+^ phase-locked mutants were cocultured with A. castellanii at an MOI of 1,000 (top) or 1 (bottom) in PYG (top) or HG (bottom) with (top) or without (bottom) 40 μg/mL Alexa Fluor-labeled dextran. Images were taken after 3 h (top) and 7 days (bottom) of coculture. The white arrows indicate GFP-expressing Bvg^+^ phase-locked B. bronchiseptica in vacuoles filled with labeled dextran. Bar, 10 μm.

We next investigated the intracellular localization of mutants deficient in adhesins in A. castellanii by microscopy. At 3 h postinfection, Bvg^+^ Δ*fhaB*, Bvg^+^ Δ*fimBCD*, and Bvg^+^ Δ*fhaB*/Δ*fimBCD* strains did not colocalize with dextran, whereas the isogenic Bvg^+^ phase-locked strain was accumulated in the dextran-filled FV of amoebae (Fig. 5). In addition, Bvg^+^ Δ*fhaB*, Bvg^+^ Δ*fimBCD*, and Bvg^+^ Δ*fhaB*/Δ*fimBCD* strains, but not the isogenic Bvg^+^ phase-locked strain, were observed within CVs after 7 days of coculture. These results indicate that *fhaB* and/or *fimBCD* Bvg^+^ phase-locked mutants enter CVs and evade A. castellanii predation, similar to WT.

## DISCUSSION

Since encountering protozoan predators that share their habitats is inevitable, B. bronchiseptica is assumed to have a means of evading predation. Indeed, the bacteria were recently shown to survive within and disseminate from soil-living D. discoideum (10); however, their survival mode in natural environments and the underlying mechanisms have remained elusive. In the present study, we investigated mutual interactions between B. bronchiseptica and A. castellanii, which has a simpler lifestyle than D. discoideum and was previously shown to serve as an environmental reservoir and nutritional source for amoeba-resistant bacteria (18, 19). We observed that the total CFU of B. bronchiseptica was significantly increased after coculture with A. castellanii (Fig. 1A and D), indicating that the amoebae may facilitate bacterial multiplication by providing nutritional sources for the bacteria through cell debris or metabolic end products. Furthermore, we consistently recovered intracellular bacteria during coculture, and the proportion of the bacteria recovered from within the amoebae was increased over time (Fig. 1D), indicating that B. bronchiseptica utilizes the amoebae as a niche for proliferation.

Previous studies reported that various bacterial species survive and/or multiply in amoeba hosts by various strategies. In order to evade digestion, Vibrio mimicus resides in the cytoplasm of A. castellanii trophozoites (20), Legionella pneumophila and Mycobacterium avium prevent phagosome-lysosome fusion (21, 22), and Burkholderia cepacia survives within the acidic vacuoles of Acanthamoeba polyphaga by an unknown mechanism (23). Although it is possible that B. bronchiseptica may localize in the amoeba cytoplasm, the present study showed that the majority of the bacteria immediately after internalization are localized in vacuoles distinct from those in the digestive pathway (Fig. 2A and see Fig. S1 in the supplemental material), which are commonly transferred to degradative phagolysosomes, suggesting that B. bronchiseptica may interfere with phagosome maturation or phagosome-lysosome fusion to evade predation by A. castellanii. Subsequently, B. bronchiseptica was localized within CVs and eventually expelled to the extracellular milieu, indicating that CVs provide a safe niche for the bacteria (Fig. 2B and C). Although experimental evidence is yet to be obtained, the bacteria are unlikely to proliferate within CVs, which periodically collapse in a period shorter than the bacterial doubling time (2, 12). Previously, V. cholerae and Salmonella enterica serovar Typhimurium were reported to localize the CVs of A. castellanii and *A. polyphaga*, respectively. V. cholerae enters CVs through vacuolar fusion between V. cholerae-containing vacuoles and CVs (11), whereas Salmonella Typhimurium enters CVs through pores on the CV membrane during fluid expulsion (24). However, the pathway through which B. bronchiseptica enters CVs remains to be determined, and we could not capture the moment of the bacterial entrance into CVs through time-lapse imaging.

Taylor-Mulneix et al. showed that B. bronchiseptica in the Bvg^−^ phase predominantly survives in the fruiting bodies of D. discoideum (10). Similarly, we showed that the Bvg^+^ phase-locked mutant of B. bronchiseptica had poorer survival in coculture with A. castellanii than WT or the Bvg^−^ phase-locked mutant (Fig. 3). Furthermore, we found that Bvg^+^ phase-locked mutants deficient in FHA and/or FIM survived, similar to WT and the Bvg^−^ phase-locked mutant, indicating that the amoebae prey on the bacteria by targeting FHA and FIM, which are the major adhesins produced by B. bronchiseptica in the Bvg^+^ phase (Fig. 4 and 5). In other words, B. bronchiseptica turns into the Bvg^−^ phase at temperatures of the natural habitats of the amoebae and evades amoeba predation by concealing FHA and FIM.

*Bordetella* FHA and FIM are adhesion molecules through which the bacteria colonize the upper respiratory tract of mammalian hosts (16, 25). FHA recognizes various host factors to initiate bacterial attachment via four distinct domains, a heparin-binding domain (HBD), an Arg-Gly-Asp (RGD) motif-containing domain, a carbohydrate recognition domain (CRD), and a mature C-terminal domain (MCD): the HBD interacts with sulfated glycoproteins and glycolipids; the RGD domain binds to three different mammalian integrins, CD11b/CD18 or αMβ2, CD51/CD61 or αVβ3, and CD49e/CD29 or α5β1 (also known as very late antigen-5 [VLA-5]); and the CRD interacts with lactose- or galactose-containing glycoconjugates (26–30). As for the MCD, although its ligands remain unknown at molecular levels, it plays a crucial role in bacterial attachment to host cells (31). FIM, which consists of several components, is also reported to recognize sulfated sugars and α5β1/VLA-5 integrin (32, 33). We speculate that FHA and FIM may interact with the above-mentioned ligands of the amoeba and facilitate bacterial uptake and digestion by amoeba cells. To verify this hypothesis, we are screening the amoeba ligands for these adhesins, the results of which may provide insight into how amoebae distinguish prey bacteria. Previous studies indicate that FHA is partly liberated from the bacterial surface (34, 35); however, only bacterium-associated FHA likely triggers the amoeba predation because only B. bronchiseptica WT survived and entered CVs when WT and the Bvg^+^ phase-locked mutant were simultaneously cocultivated with the amoebae (Fig. S3). In addition, the Bvg^+^ phase-locked Δ*fhaB*/Δ*fimBCD* mutant was recovered in higher numbers from coculture with A. castellanii or D. discoideum than was the single knockout Δ*fhaB* or Δ*fimBCD* mutant, indicating that FHA and FIM are partially redundant targets for amoeba predation (Fig. 4D). Hence, amoebae may recognize prey bacteria using amoeba factors like α5β1/VLA-5 integrin or sulfated sugars, which commonly function as ligands for FHA and FIM.

In contrast to B. bronchiseptica, B. pertussis was not recovered from amoebae after 7 days of coculture, even though the number of extracellular bacteria was significantly increased (Fig. 1C). Similar results were previously reported by Ma et al.; B. pertussis was not recovered from the cells or sori of D. discoideum (36). B. pertussis has the ability for Bvg-phase conversion and produces orthologous FHA and FIM in the Bvg^+^ phase. Study of why B. pertussis cannot survive in amoebae will assist in clarifying the survival mechanism of B. bronchiseptica. A previous report demonstrated that at 24°C, B. pertussis maintained the production of virulence factors, including FHA (37). B. pertussis, which produces FHA and probably FIM at standard temperatures used for coculture with amoebae, may be preyed upon by the amoebae. However, this was not the case in our experimental design because FHA- and FIM-deficient B. pertussis did not survive within A. castellanii (data not shown). These results imply an additional mechanism other than FHA and FIM sequestration for the intra-amoeba survival of B. bronchiseptica. B. pertussis has lost a large number of genes during adaptation to a closed life cycle in humans (36, 38). It is likely that Bvg^−^-specific factors produced by B. bronchiseptica but not by B. pertussis due to these genetic deletions may contribute to evading amoeba predation in addition to FHA and FIM sequestration.

There is a consensus that the Bvg-phase conversion of B. bronchiseptica is necessary for its wide range of life cycles between intra- and extrahost environments. In particular, the Bvg^−^ phase is considered important for survival in extrahost environments. Our present study provides a molecular basis for this phenomenon. Future studies on the behavior of B. bronchiseptica in natural environments, including interactions with protists, may allow us to further understand the biological significance of the Bvg phases of *Bordetella* spp.

## MATERIALS AND METHODS

### Bacterial strains.

All bacterial strains and plasmids used in this study are listed in Table S1 in the supplemental material. B. bronchiseptica strain RB50 was provided by Peggy A. Cotter (University of North Carolina, USA), B. bronchiseptica strain S798 was provided by Nobuyuki Terakado (National Institute of Animal Health, Japan), and B. pertussis strain Tohama (39) was maintained in our laboratory. *Bordetella* strains were grown at 37°C for 2 to 3 days on Bordet-Gengou (BG) agar plates (Becton, Dickinson) containing 1% Hipolypeptone (Nihon Pharmaceutical), 1% glycerol, 15% defibrinated horse blood, and 10 μg/mL ceftibuten. The bacteria grown on BG plates were suspended in Stainer-Scholte (SS) broth (40, 41) to make a value of optical density at 650 nm (OD_650_) of 0.1 (B. pertussis) or 0.02 (B. bronchiseptica) and incubated at 37°C with shaking for 16 h. The CFU was estimated from the OD_650_ values of fresh cultures according to the following equation: 1 OD_650_ unit = 3.3 × 10^9^ CFU/mL. Escherichia coli was grown at 37°C on Luria-Bertani (LB) agar or in LB broth. When necessary, the growth media were supplemented with antibiotics at the following concentrations: 10 μg/mL gentamicin (Gm) and 30 μg/mL kanamycin (Km).

### Construction of bacterial mutant strains.

The primers used for construction of the bacterial mutant strains are listed in Table S2. Fluorescent protein-expressing B. bronchiseptica strains were generated as follows. A *tac* promoter-driven *gfp* gene, which was amplified by PCR using pBBR1MCS5-P*tac*-GFP (42) as a template with the primers BD42-F and BD43-R, was inserted 33 bp downstream of the Km resistance gene (Km^r^) in the plasmid pMariK harboring the *mariner* transposon (43) using an In-Fusion HD cloning kit (Clontech). The resultant plasmid, pMariK-GFP, was introduced into E. coli S17-1 λpir and transconjugated into B. bronchiseptica RB50 by biparental conjugation. Transposon-based integration of the Km^r^-P*tac*-GFP fragment into the B. bronchiseptica genome was confirmed by colony PCR using the primers BS333-F and BS334-R. The integration site of Km^r^-P*tac*-GFP was determined as previously described (44). Briefly, genomic DNA (gDNA) of a single mutant was isolated using the DNeasy Blood and Tissue kit (Qiagen) and digested with Sau3AI (TaKaRa). The digested products were self-ligated with T4 DNA ligase (Promega) and used as the templates for inverse PCR using the primers Mari5 and Mari7. The PCR products were subjected to direct sequencing using the same primers, and the obtained sequences were aligned to B. bronchiseptica RB50 genome (NCBI accession no. NC_002927.3) as a reference to identify the transposon insertion site. We selected a single clone of the transposon-inserted mutant, in which the *mariner* transposon was incorporated in nucleotide position 762 of the *BB4978* gene of B. bronchiseptica, which encodes a hypothetical protein with an unknown function (Fig. S5A). There was no growth difference between WT and the GFP-expressing mutant at 37°C and 20°C, indicating that integration of the *mariner* transposon into the *BB4978* gene did not affect bacterial viability (Fig. S5B). Even after prolonged incubation for 144 h at 20°C in HG, the level of GFP expression of the bacterium was unchanged (Fig. S5C).

mCherry-expressing B. bronchiseptica was constructed by integrating the Km^r^-P*tac*-mCherry fragment into the same site of the bacterial genome where the transposon carrying the Km^r^-P*tac*-GFP fragment was integrated (nucleotide position 762 of BB4978). Briefly, P*tac*-mCherry from pBBR1MCS5-P*tac*-mCherry (lab collection) was amplified using the primers BD216-F and BD217-R and inserted 33 bp downstream of the Km resistance gene (Km^r^) in plasmid pMariK. The resulting plasmid was designated pMariK-mCherry. A suicide plasmid for knock-in was constructed using the plasmid pABB-CRS2-Gm (45). A 2.2-kbp DNA fragment from *BB4977* to *BB4979* genes was amplified by PCR using gDNA of B. bronchiseptica RB50 as the template and the primers BD168-F and BD169-R. The fragment was then ligated into the SmaI site 14 bp upstream of the *lac* operator in plasmid pABB-CRS2-Gm, resulting in pABB-CRS2-Gm-RB50-BB4978. Linearized pABB-CRS2-Gm-RB50-BB4978 was obtained by inverse PCR using the primers BD222-F and BD223-R. Km^r^-P*tac*-mCherry without mariner repeat (MR) from pMariK-mCherry plasmid was amplified using the primers BD224-F and BD225-R and ligated to linearized pABB-CRS2-Gm-RB50-BB4978 (at nucleotide position 762 of BB4978). The resulting plasmid, designated pABB-CRS2-Gm-RB50-BB4978-mCherry, was introduced into E. coli S17-1 λpir and transconjugated into B. bronchiseptica RB50 by biparental conjugation. The Km^r^-P*tac*-mCherry gene was inserted into the genome of B. bronchiseptica RB50 by a two-step homologous recombination through counterselection with 10% sucrose.

Gene deletion strains were constructed as described previously (46). For example, a *fimBCD*-deficient mutant (RB50 Δ*fimBCD*) was generated as follows. Approximately 1,000 bp of the 5′- and 3′-flanking regions of the *fimBCD* gene (named *fimBCD*-upstream and *fimBCD*-downstream, respectively) was amplified by PCR with B. bronchiseptica RB50 gDNA as a template and primer combinations of fimBCD-US and fimBCD-UAS and of fimBCD-DS and fimBCD-DAS. The amplified fragments were ligated to each other by overlap PCR via an overlapping region of the primers (Table S2, boldface) and inserted into the SmaI site 14 bp upstream of the *lac* operator in plasmid pABB-CRS2-Gm using an In-Fusion HD cloning kit (Clontech). The resultant suicide plasmid, pABB-CRS2-Gm-Δ*fimBCD*, was introduced into E. coli S17-1 λpir and transconjugated into B. bronchiseptica by biparental conjugation. The target gene was deleted by a two-step homologous recombination using 10% sucrose for counterselection.

The Bvg^−^ phase-locked mutant in strain RB50 and Bvg^+^ phase-locked mutants in strains RB50 and S798 were constructed as previously described with slight modifications (42, 47). The *bvgS* gene was amplified by PCR with B. bronchiseptica RB50 gDNA using the primers BD117-F and BD120-R. The resultant fragment was inserted into the SmaI site 14 bp upstream of the *lac* operator in pABB-CRS2-Gm, and the plasmid was designated pABB-CRS2-Gm-*bvgS*_full_. Inverse PCR was carried out with the primers BD118-R and BD119-F and pABB-CRS2-Gm-*bvgS*_full_ as a template, which resulted in the deletion of the gene corresponding to amino acids (aa) 541 to 1020 of BvgS. The PCR products were 5′ phosphorylated with T4 polynucleotide kinase (TaKaRa) and self-ligated with T4 DNA ligase (Promega). The resultant plasmid was designated pABB-CRS2-Gm-Δ*bvgS* and used to generate the Bvg^−^ phase-locked strain.

The *bvgS*-C3 mutation (replacement of Arg with His at aa 570) was introduced into the pABB-CRS2-Gm-*bvgS*_full_ by PCR using the primers BD121-F and BD122-R. The resultant plasmid was designated pABB-CRS2-Gm-*bvgS*-C3 and used to generate the Bvg^+^ phase-locked strains. The plasmids pABB-CRS2-Gm-Δ*bvgS* and pABB-CRS2-Gm-*bvgS*-C3 were respectively introduced into E. coli S17-1 λ*pir* and transconjugated into B. bronchiseptica by biparental conjugation. The mutation (G-to-A change at nucleotide position 1709) and deletion of the *bvgS* gene in the resultant Bvg^+^ and Bvg^−^ phase-locked mutants were confirmed by sequencing.

### Coculture conditions for B. bronchiseptica and A. castellanii.

The peptone-yeast-glucose (PYG) medium, which is widely used for the cultivation of amoebae (48), is unsuitable for coculture assay. B. bronchiseptica was found to proliferate in PYG medium after a 4-day incubation at 20°C (Fig. S6A), which may lead to biased interpretations as to whether the bacteria grow by the aid of amoebae. Thus, searching for a suitable coculture medium in which B. bronchiseptica cannot grow but remains viable upon prolonged incubation at 20°C is necessary. The medium also should support the viability of A. castellanii in the metabolically active trophozoite form. In addition, the medium should be isotonic with the intracellular solute concentration and contain a carbon source. In search of such media, various concentrations of glucose (0, 5, 20, and 80 mM) as the carbon source and sodium chloride (NaCl) (0, 80, and 135 mM) as the osmolyte and inorganic ion source (49) in 50 mM HEPES buffer, pH 7.4, were examined. The A. castellanii suspension was seeded at a density of 1 × 10^5^ cells into each well of a 24-well plate containing 50 mM HEPES buffer (Sigma-Aldrich), pH 7.4, supplemented with different concentrations of glucose and NaCl (Fig. S6B). The culture plates were incubated at 20°C, and A. castellanii morphology was observed at day 7 of incubation. A. castellanii encysted at higher concentrations of glucose and NaCl or no glucose but remained in the trophozoite form at 5 mM glucose (Fig. S6B, red box). Furthermore, bacteria were viable but did not proliferate at 5 mM glucose (data not shown). Therefore, the coculture assay was performed in 50 mM HEPES buffer, pH 7.4, containing 5 mM glucose (here referred to as HG).

### Amoeba culture conditions.

A. castellanii strain C3 (ATCC 50739) was maintained axenically in PYG broth at 20°C as described previously (50) in a T-25 culture flask (CellStar) without shaking. Dictyostelium discoideum strain AX2 (National BioResource Project, identifier [ID] S00001) was grown in HL5 medium at 20°C (51). After a 4- to 6-day incubation, the grown amoebae were detached by vigorous shaking of the flasks and centrifuged at 1,200 rpm for 3 min at room temperature (RT). After discarding the supernatant, the amoeba pellets were suspended in HG. The viability of the amoebae was confirmed by the dye-exclusion test with 0.2% trypan blue. The numbers of amoeba trophozoites were enumerated using a Bürker-Türk-type cell-counting chamber (Watson).

### Enumeration of B. bronchiseptica after coculture with amoebae and A. castellanii viability test.

The ability of bacteria to survive in A. castellanii trophozoites was evaluated using GPA as described previously (10). Briefly, amoebae (2 × 10^5^ cells) were cocultured with B. bronchiseptica (2 × 10^7^ CFU) in 24-well plates. The bacteria and amoebae in the wells were settled by centrifugation (200 × *g*, 10 min, RT) and incubated at 20°C to allow amoeba-bacterium interactions. After 1 h, the bacteria and amoebae in the wells were treated with 300 μg/mL of Gm (Nacalai Tesque) in PYG, followed by further incubation for 3 h at 20°C to kill extracellular bacteria. Antibiotic traces were eliminated by at least two washes with phosphate-buffered-saline (PBS; 137 mM NaCl, 2.7 mM KCl, 8 mM Na_2_HPO_4_, 1.47 mM KH_2_PO_4_). Amoeba cells were lysed with 200 μL of 0.1% Triton X-100 (Nacalai Tesque) at 20°C for 10 min. The lysed amoeba suspension was serially diluted with PBS and spread on BG agar plates. The bacteria on the plates were incubated at 37°C for 2 days, and the CFU was enumerated. The phenotypes of the Bvg phase-locked mutants recovered from the amoebae were confirmed by colony appearance after incubation at 37°C for the Bvg^−^ phase-locked mutant and at 20°C for the Bvg^+^ phase-locked mutant: the Bvg^−^ phenotype exhibits large, flat, and nonhemolytic colonies while the Bvg^+^ phenotype exhibits small, domed, and hemolytic colonies.

The long-term survival of B. bronchiseptica in the presence of amoebae was evaluated using a coculture assay. A. castellanii or D. discoideum seeded in one well of a 24-well plate at 1 × 10^5^ cells was infected with B. bronchiseptica at a multiplicity of infection (MOI) of 1 in HG and incubated at 20°C. After the indicated incubation periods, the bacteria and amoebae were suspended by vigorous pipetting and flushed out through a 27-gauge syringe (Terumo) to disrupt the amoeba cells. The lysed-amoeba suspension was serially diluted with PBS and spread on BG agar plates. The bacteria on the plates were incubated at 37°C for 2 days, and the total CFU was enumerated. The intracellular bacterial number was determined after Gm treatment and cell lysis with Triton X-100 as described above. To test the effect of B. bronchiseptica on A. castellanii viability, the coculture assay was performed as described above at an MOI of 100. The amoeba suspension at the indicated periods of incubation was collected by vigorous pipetting and centrifuged at 8,000 rpm for 2 min. The pellets were resuspended in HG and aliquoted for the enumeration of trophozoites.

### Fluorescence microscopy for the localization of B. bronchiseptica in A. castellanii.

Amoebae cultivated in a 35-mm glass base dish (Eppendorf) were infected with GFP- and/or mCherry-expressing B. bronchiseptica at an MOI of 1,000 or 3,000 in 2 mL of PYG or HG with or without 40 μg/mL of Alexa Fluor 647-labeled dextran (size, 10,000 molecular weight; Invitrogen) at 20°C. After the indicated incubation periods, the specimens were examined under a confocal microscope (LSM880 Zeiss). For time-lapse imaging, pictures were taken every 15 s at selected time points. All images were processed using ImageJ software (NIH; https://imagej.nih.gov/ij/index.html).

### Reverse transcription-PCR.

Total RNA was extracted from B. bronchiseptica after overnight incubation in SS broth using a NucleoSpin RNA kit (Macherey-Nagel) and treated with RNase-free DNase I (TaKaRa) to degrade residual gDNA. Aliquots of 500 ng of total RNA were reverse transcribed into cDNA using a PrimeScript RT reagent kit (TaKaRa). PCR was performed using cDNA as the template with the primers listed in Table S2 under the following conditions: initial denaturation at 94°C for 2 min and 35 cycles of 98°C for 10 s, 58°C for 30 s, and 68°C for 11 s. The PCR products were then subjected to electrophoresis on a 2% agarose gel containing 0.005% ethidium bromide.

### Statistical analysis.

Statistical analyses were performed to evaluate differences between test groups using Prism 9 (GraphPad Software). Data were obtained from three independent experiments and are expressed as the mean ± standard deviation (SD) from triplicate experiments. Significance is expressed as *P* values (*, *P* < 0.05; **, *P* < 0.01; ***, *P* < 0.001; and ****, *P* < 0.0001).
