# Supplementary material for: Survival of Bordetella bronchiseptica in Acanthamoeba castellanii
Source: Microbiol Spectr. 2023 Mar 27;11(2):e00487-23. doi: 10.1128/spectrum.00487-23 (PMC10100856; doi:10.1128/spectrum.00487-23)
Supplement: Supplemental file 1 — Supplemental material. Download spectrum.00487-23-s0001.pdf, PDF file, 2.6 MB [file spectrum.00487-23-s0001.pdf]

SUPPLEMENTAL MATERIAL

Survival of *Bordetella bronchiseptica* in *Acanthamoeba castellanii*

Dendi Krisna Nugraha[a]#, Takashi Nishida[a]#, Yuki Tamaki[a], Yukihiro Hiramatsu[a], Hiroyuki Yamaguchi[b], and Yasuhiko Horiguchi[a,c]

[a]Department of Molecular Bacteriology, Research Institute for Microbial Diseases, Osaka University, 3-1 Yamada-oka, Suita, 565-0871, Osaka, Japan

[b]Department of Medical Laboratory Science, Faculty of Health Sciences, Hokkaido University, Kita 12 Nishi 5 Kita-ku, Sapporo, 060-0812, Hokkaido, Japan

[c]Center for Infectious Disease Education and Research, Osaka University, 2-8 Yamada-oka, Suita, 565-0871, Osaka, Japan

#Corresponding author: Dendi Krisna Nugraha and Takashi Nishida

TEL: +81-6-6879-8285, FAX: +81-6-6879-8283

E-mail: dendi@biken.osaka-u.ac.jp and t-nishida@biken.osaka-u.ac.jp

Supplemental Figures

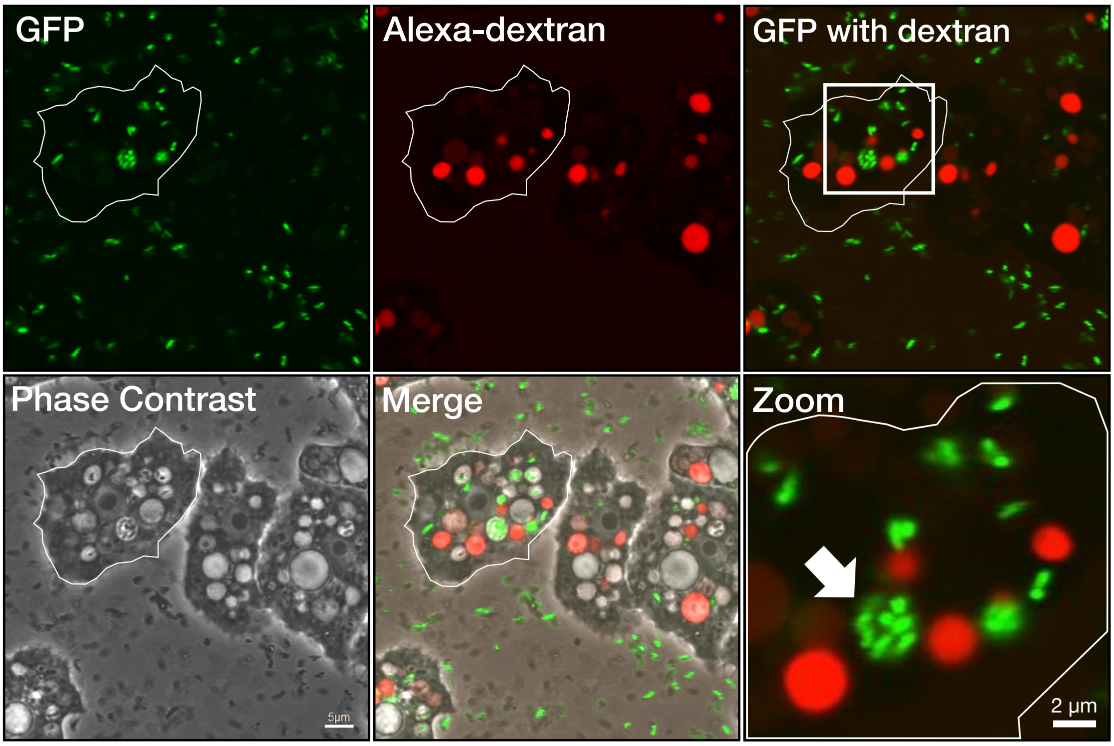

Fig. S1. Intracellular localization of GFP-expressing *B. bronchiseptica* in *A. castellanii.*
Amoeba cells were infected with bacteria (green) at an MOI of 1,000 in PYG medium containing dextran (red) for 6 h. Note that the bacteria were internalized in vacuoles that do not contain dextran (white arrow). Bar: 5 µm or 2 µm (zoom).

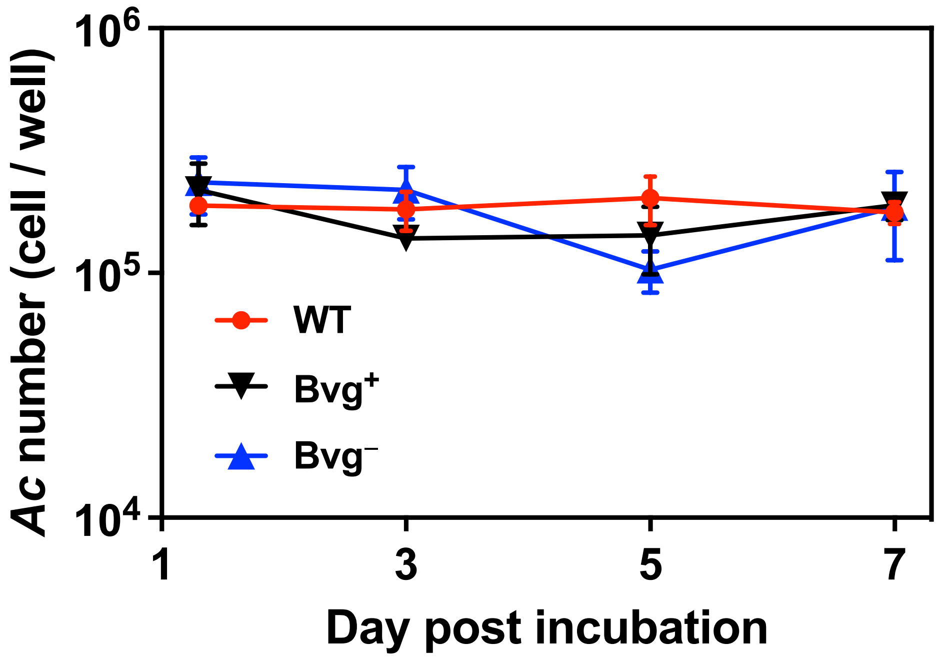

Fig. S2. The viability of *A. castellanii* in the presence of *B. bronchiseptica* WT, Bvg^+ phase-locked or Bvg^- phase-locked mutants. *A. castellanii* was infected with *B. bronchiseptica* WT, Bvg^+ phase-locked mutant, or Bvg^- phase-locked mutant at an MOI of 100, and the total amoeba cells after 1, 3, 5, and 7 days of incubation in HG were counted after 0.2% trypan blue staining. Values represent the mean ± SD (n = 3).

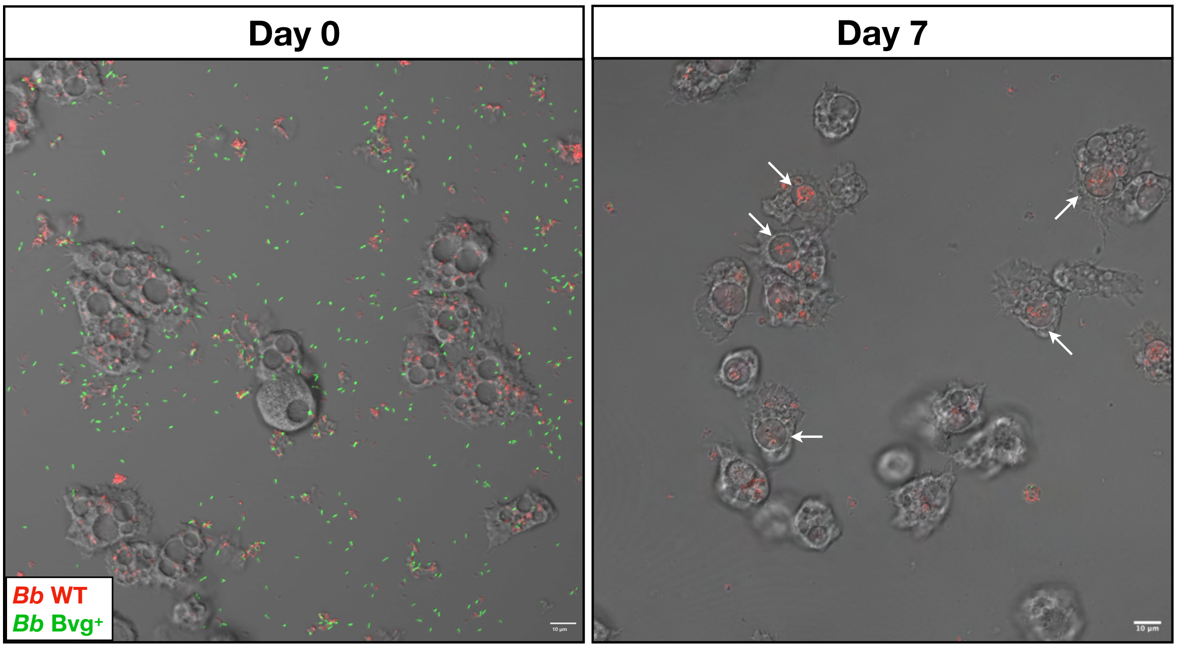

Fig. S3. Coculture of *A. castellanii with B. bronchiseptica* WT and Bvg$^+$ phase-locked mutant. mCherry-expressing *B. bronchiseptica* WT was mixed with the GFP-expressing Bvg$^+$ phase-locked mutant (ratio of 1:1) and incubated with *A. castellanii* at an MOI of 1,000. The white arrows indicate CVs filled with mCherry-expressing *B. bronchiseptica* WT. Note that only mCherry-expressing WT was localized in CVs. Bar: 10 µm.

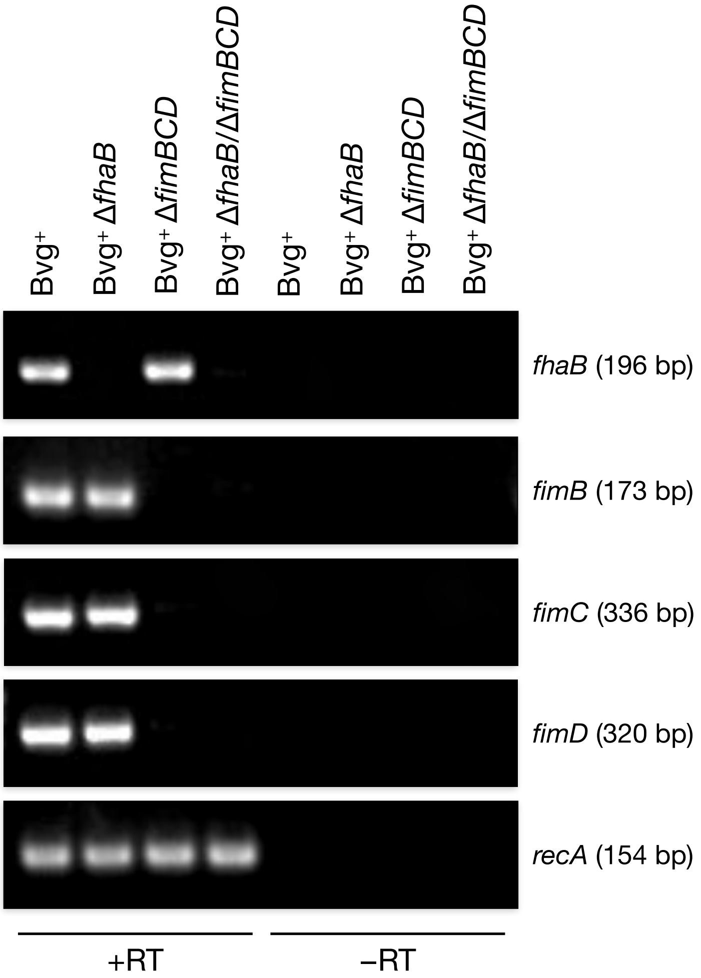

Fig. S4. Reverse transcription (RT)-PCR analysis of *fhaB*, *fimB*, *fimC*, *fimD*, and *recA* mRNA transcripts in *B. bronchiseptica* Bvg$^+$ phase-locked mutants. Total RNA of *B. bronchiseptica* Bvg$^+$ phase-locked RB50 mutant was extracted from an overnight culture grown in SS medium. RT-PCR reactions with purified RNA only (−RT) were included as negative controls. Expression of *recA* was used as the internal control for each sample. The length of the PCR products of each gene is indicated in brackets.

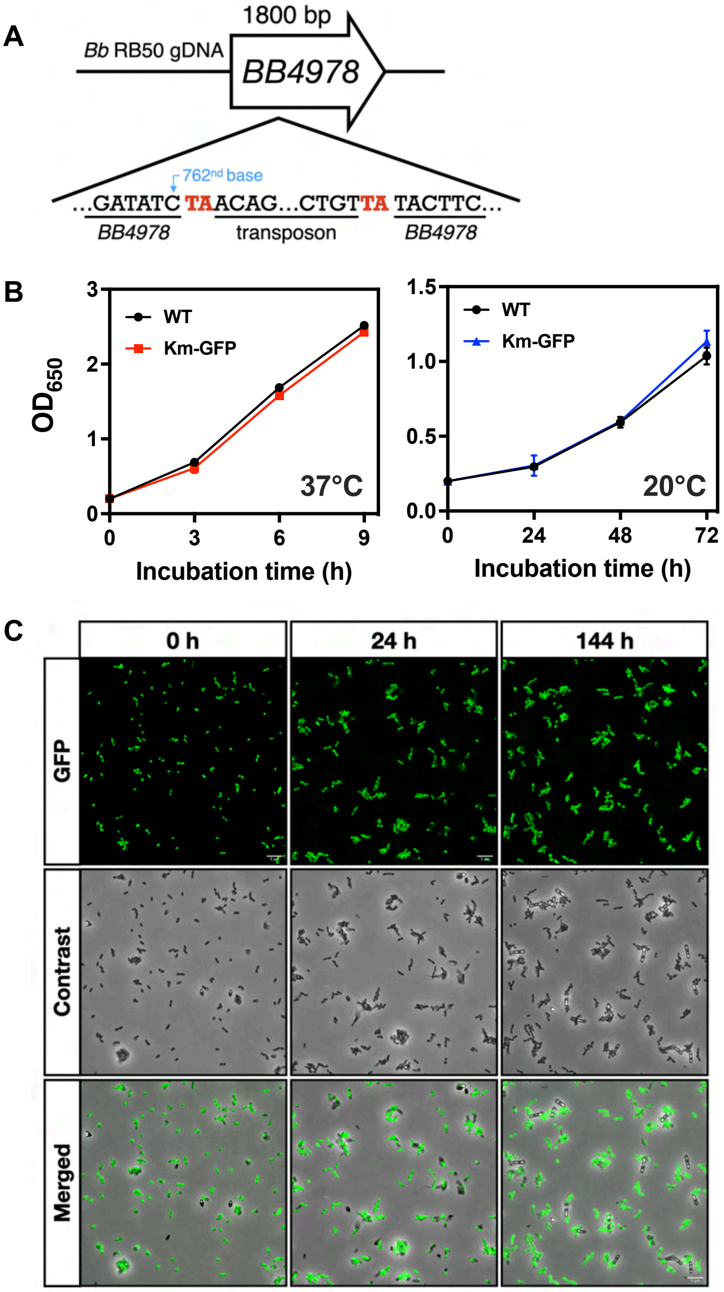

Fig. S5. Development of stable GFP-expressing *B. bronchiseptica* using a *mariner*-based transposon. (A) The transposon is inserted within the gDNA of GFP-expressing *B. bronchiseptica* (*Bb*) at nucleotide position 762 of the *BB4978* gene (corresponding to the 254th amino acid of BB4978). The target TA dinucleotides for the transposon insertion are indicated by red letters. (B) Growth of WT and GFP-expressing strains incubated in SS broth at 37°C (left) and 20°C (right). Data expressed as OD_{650} values represent the mean ± SD (n = 3). (C) GFP expression of the bacteria at 0, 24, and 144 h post incubation in HG at 20°C. Bar: 5 µm.

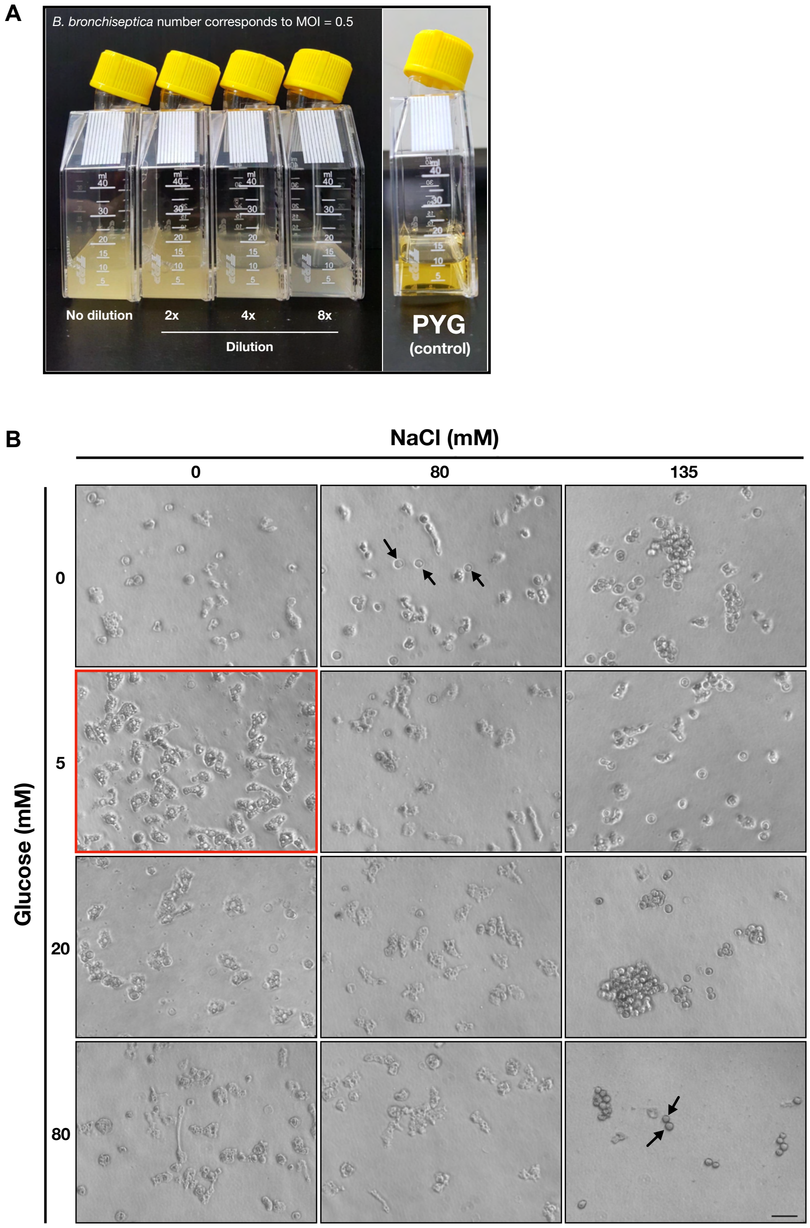

Fig. S6. HEPES-glucose medium (HG) is suitable for coculture assay with *B. bronchiseptica*. (A) *B. bronchiseptica* growth in PYG medium after 4-day incubation at 20°C. Ten milliliters of undiluted or diluted (2, 4, and 8x) PYG in T-25 flasks were inoculated with ~10^4 CFU of *B. bronchiseptica*. T-25 flask containing only PYG medium served as the control. (B) *A. castellanii* cultured in 50 mM HEPES buffer (pH 7.4) containing glucose and NaCl. Amoebae (1×10^5 cells) were seeded into each well containing HEPES buffer (pH 7.4) supplemented with different concentrations of glucose (0, 5, 20, and 80 mM) and NaCl (0, 80, and 135 mM). After incubation at 20°C for 7 days, images from each well were captured. Black arrows indicate amoeba cysts. Bar: 20 µm.

Supplemental Video Legend 1

Movie S1. Time-lapse imaging of intracellular *B. bronchiseptica* escaping from CV (also shown in Fig. 2C).

Supplemental Table 1. List of bacterial strains and plasmids used in this study

Strains and plasmids	Description	Source
B. bronchiseptica		
RB50	WT, Isolated from rabbit	P. A. Cotter
RB50 BB4978::KmR-GFP	RB50 derivative	This study
RB50 BB4978::KmR-GFP Bvg$^+$ phase-locked	RB50 derivative	This study
RB50 BB4978::KmR-GFP Bvg$^-$ phase-locked	RB50 derivative	This study
RB50 BB4978::KmR-mCherry	RB50 derivative	This study
RB50 Bvg$^-$ phase-locked	RB50 derivative	This study
RB50 Bvg$^+$ phase-locked	RB50 derivative	This study
RB50 Δ*fhaB* Bvg$^+$ phase-locked	RB50 derivative	This study
RB50 Δ*fimBCD* Bvg$^+$ phase-locked	RB50 derivative	This study
RB50 Δ*fhaB*Δ*fimBCD* Bvg$^+$ phase-locked	RB50 derivative	This study
S798	WT, Clinical isolate	(1)
S798 Δ*bscN*Δ*dnt*Δ*prn*Δ*cyaA* (Δ4) Bvg$^+$ phase-locked	S798 derivative	This study
S798 Δ4 Δ*fhaB* (Δ5) Bvg$^+$ phase-locked	S798 derivative	This study
S798 Δ5 Δ*fimBCD* (Δ6) Bvg$^+$ phase-locked	S798 derivative	This study
S798 Δ6 Δ*vag8* (Δ7) Bvg$^+$ phase-locked	S798 derivative	This study
S798 Δ7 Δ*fhaS* (Δ8) Bvg$^+$ phase-locked	S798 derivative	This study
S798 Δ8 Δ*fhaL* (Δ9) Bvg$^+$ phase-locked	S798 derivative	This study
S798 Δ9 Δ*brtA* (Δ10) Bvg$^+$ phase-locked	S798 derivative	This study
B. pertussis		
Tohama I	WT, Vaccine strain	(2)
E. coli		
S17-1 λpir	K-12 strain for plasmid with R6K origin	Lab collection
**Plasmids**		
pBBR1MCS5-P*tac*-GFP	pBBR1MCS5 carrying *tac* promoter, *gfp* and *trpA* terminator	(3, 4)
pBBR1MCS5-P*tac*-mCherry	pBBR1MCS5 carrying *tac* promoter, *mCherry* and *trpA* terminator	Lab collection
pMariK	Mariner transposon vector carrying KmR	(5)
pMariK-GFP	pMariK carrying *tac* promoter, *gfp* and *trpA* terminator	This study
pMariK-mCherry	pMariK carrying *tac* promoter, *gfp* and *trpA* terminator	This study
pMariG	Mariner transposon vector carrying GmR	(6)
pABB-CRS2-Gm	GmR, R6K-derived suicide vector	(7)
pABB-CRS2-Gm-RB50-BB4978	*BB4978* fragment cloned into pABB-CRS2-Gm	This study
pABB-CRS2-Gm-RB50-BB4978-mCherry	P*tac*-mCherry fragment at *BB4978* site cloned into pABB-CRS2-Gm	This study
pABB-CRS2-Gm-Δ*fhaB*	*fhaB* deletion cloned into pABB-CRS2-Gm	This study
pABB-CRS2-Gm-Δ*fimBCD*	*fimBCD* deletion cloned into pABB-CRS2-Gm	This study
pABB-CRS2-Gm-$bvgS_{\text{full}}$	*bvgS* fragment cloned into pABB-CRS2-Gm	This study
pABB-CRS2-Gm-Δ*bvgS*	*bvgS* deletion cloned into pABB-CRS2-Gm ($\Delta_{541\text{-}1020}$)	This study
pABB-CRS2-Gm-*bvgS*-C3	*bvgS*-C3 mutation cloned into pABB-CRS2-Gm (R570H)	This study
pABB-CRS2-Gm-Δ*bscN* (S798)	*bscN* deletion cloned into pABB-CRS2-Gm for Δ1 construction	Lab collection
pABB-CRS2-Gm-Δ*dnt* (S798)	*dnt* deletion cloned into pABB-CRS2-Gm for Δ2 construction	Lab collection
pABB-CRS2-Gm-Δ*prn* (S798)	*prn* deletion cloned into pABB-CRS2-Gm for Δ3 construction	Lab collection
pABB-CRS2-Gm-Δ*cyaA* (S798)	*cyaA* deletion cloned into pABB-CRS2-Gm for Δ4 construction	Lab collection
pABB-CRS2-Gm-Δ*fhaB* (S798)	*fhaB* deletion cloned into pABB-CRS2-Gm for Δ5 construction	Lab collection
pABB-CRS2-Gm-Δ*fimBCD* (S798)	*fimBCD* deletion cloned into pABB-CRS2-Gm for Δ6 construction	Lab collection
pABB-CRS2-Gm-Δ*vag8* (S798)	*vag8* deletion cloned into pABB-CRS2-Gm for Δ7 construction	Lab collection
pABB-CRS2-Gm-Δ*fhaS* (S798)	*fhaS* deletion cloned into pABB-CRS2-Gm for Δ8 construction	Lab collection
pABB-CRS2-Gm-Δ*fhaL* (S798)	*fhaL* deletion cloned into pABB-CRS2-Gm for Δ9 construction	Lab collection
pABB-CRS2-Gm-Δ*brtA* (S798)	*brtA* deletion cloned into pABB-CRS2-Gm for Δ10 construction	Lab collection

Supplemental Table 2. List of primers used in this study

Name	Sequence (5' to 3')	Application
BD41-F	CCAATTCTGATTAGAAAAACTCATCGA	Construction of pMariK-GFP
BS179-R	TTAATTGGTTGTAACACTGGCAGAG	
BD42-F	TTTCTAATCAGAATTGGGGCAAATATTCTGAAATGAGC	
BD43-R	TGTTACAACCAATTAATTCTCCTACATGTTCGCC	
BD216-F	CGATGAGTTTTTCTAATCAGAATTGGTGCGCCGACATCATAACGG	Construction of pMariK-mCherry
BD217-R	CTGCCAGTGTTACAACCAATTAACGGTTCTCCTACATGTTCGCCT	
BD85-R	GGGAGAGCTCGGATCCACTAGT	Construction of pABB-CRS2-Gm-RB50-BB4978
BD86-F	GGGAATTCCACAAATTGTTATCCGCT	
BD168-F	AGTGGATCCGAGCTCTCCCACCGTTCGAACCGCTGGATAT	
BD169-R	AACAATTTGTGGAATTCCCAATCGTCGCCGTAATTGCGT	
BD222-F	TTCCAGGGTTACGCCAACCG	Construction of pABB-CRS2-Gm-RB50-BB4978-mCherry
BD223-R	GATATCCTCGTGCATGGCCTCG	
BD224-F	AGGCCATGCACGAGGATATCATGGTCTAGAGTCGACCTCGAGG	
BD225-R	CGGTTGGCGTAACCCTGGAACGGTTCTCCTACATGTTCGCCT	
Mari5	GACTCTAGACCATGGTTGTGTC	Determination of transposon insertion site
Mari7	ATGCCTCTTCCGACCATCAAGC	
BS333-F	GACACAACCATGGTCTAGAGTCGACCTC	Colony PCR to check P*tac*-GFP integration
BS334-R	CCCTTCAAGAGCGATACCCGAGC	
fhaB-US	TAGTGGATCCGAGCTCTCCCGTGAAAAGAAAGAAATGGAAAACAA	Construction of pABB-CRS2-Gm-Δ*fhaB* (RB50)
fhaB-UAS	**TATGAAACCAACAAA**TAGGTAGTCGCTGCC	
fhaB-DS	**TTTGTTGGTTTCATA**GCCCGCACACGCCAA	
fhaB-DAS	TAACAATTTGTGGAATTCCCGCGTACTGTCGCTCTTGGCG	
fimBCD-US	TAGTGGATCCGAGCTCTCCCGAAACTGCGCGAA	Construction of pABB-CRS2-Gm-Δ*fimBCD* (RB50)
fimBCD-UAS	**GTCGACAGTACTCTCGA**GACCTTGAACTATGACTGACG	
fimBCD-DS	**TCGAGAGTACTGTCGA**CACAAGCGTATGTATTCATG	
fimBCD-DAS	TAACAATTTGTGGAATTCCCATTGCAGCCCATCCTTT	
BD117-F	TAGTGGATCCGAGCTCTCCCACGCTGCATTACTTCCCATC	Construction of pABB-CRS2-Gm-*bvgS*$_{full}$ (RB50)
BD120-R	AACAATTTGTGGAATTCCCTTACCGTCAGTACGTTCGATG	
BD118-R	CTCGTTGCGGTAGGCGTA	Construction of pABB-CRS2-Gm-Δ*bvgS* (RB50)
BD119-F	ATCACCGATTGCAACATGCC	
BD121-F	GGCGCCAGATCCGCCAGCACAAGCGGGCCGAGCGGG	Construction of pABB-CRS2-Gm- *bvgS*-C3 (RB50 and S798)
BD122-R	CCGCTCGGCCCGCTTGTGCTGGCGGATCTGGCGCC	
BD218-F	TAGTGGATCCGAGCTCTCCCACGCTGCATTACTTCCCATCAT	Construction of pABB-CRS2-Gm-*bvgS*$_{full}$ (S798)
BD219-R	ATAACAATTTGTGGAATTCCCTCAGGTCCACCGAAACCGTT	
BD323-F	TTTCGTCCACTACGCTGGC	RT-PCR for *fhaB*
BD324-R	TGCTTGTTGCGCTGAACG	
BD302-F	GAGACCACGGTGAAGATGAGC	RT-PCR for *fimB*
BD303-R	TTGAAGAAAATGCGCAAGGTCTG	
BD325-F	AGCCGATTCAACTACAGCAACG	RT-PCR for *fimC*
BD326-R	GTTGATCGTATAGGCCTGCGGA	
BD293-F	TGCCGTTCTGGTGCTATGG	RT-PCR for *fimD*

BD287-R	GTCATAGTTCAAGGTCACGGCGTAGGT	
recA-F	GCTGACTATGCCCTGGTTCT	RT-PCR for *recA*
recA-R	AGCCAATGTGGTCGACAAGT	

Supplemental References

1. Okada K, Ogura Y, Hayashi T, Abe A, Kuwae A, Horiguchi Y, Abe H. 2014. Complete genome sequence of *Bordetella bronchiseptica* S798, an isolate from a pig with atrophic rhinitis. *Genome Announc* 2:e00436-14.
2. Parkhill J, Sebaihia M, Preston A, Murphy LD, Thomson N, Harris DE, Holden MTG, Churcher CM, Bentley SD, Mungall KL, Cerdeño-Tárraga AM, Temple L, James K, Harris B, Quail MA, Achtman M, Atkin R, Baker S, Basham D, Bason N, Cherevach I, Chillingworth T, Collins M, Cronin A, Davis P, Doggett J, Feltwell T, Goble A, Hamlin N, Hauser H, Holroyd S, Jagels K, Leather S, Moule S, Norberczak H, O'Neil S, Ormond D, Price C, Rabbinowitsch E, Rutter S, Sanders M, Saunders D, Seeger K, Sharp S, Simmonds M, Skelton J, Squares R, Squares S, Stevens K, Unwin L, Whitehead S, Barrell BG, Maskell DJ. 2003. Comparative analysis of the genome sequences of *Bordetella pertussis*, *Bordetella parapertussis* and *Bordetella bronchiseptica*. *Nat Genet* 35:32–40.
3. Kovach ME, Elzer PH, Hill DS, Robertson GT, Farris MA, Roop RM, Peterson KM. 2015. Four new derivatives of the broad-host-range cloning vector pBBR1MCS, carrying different antibiotic-resistance cassettes. *Gene* 166:1–2.
4. Nishikawa S, Shinzawa N, Nakamura K, Ishigaki K, Abe H, Horiguchi Y. 2016. The Bvg-repressed gene *brtA*, encoding biofilm-associated surface adhesin, is expressed during host infection by *Bordetella bronchiseptica*. *Microbiol Immunol* 60:93–105.
5. Tsukamoto K, Shinzawa N, Kawai A, Suzuki M, Kidoya H, Takakura N, Yamaguchi H, Kameyama T, Inagaki H, Kurahashi H, Horiguchi Y, Doi Y. 2020. The *Bartonella* autotransporter BafA activates the host VEGF pathway to drive angiogenesis. *Nat Commun* 11:3571.
6. Hiramatsu Y, Nishida T, Nugraha DK, Sugihara F, Horiguchi Y. 2021. Melanin produced by *Bordetella parapertussis* confers a survival advantage to the bacterium during host infection. *mSphere* 6:e00819-21.
7. Sekiya K, Ohishi M, Ogino T, Tamano K, Sasakawa C, Abe A. 2001. Supermolecular structure of the enteropathogenic *Escherichia coli* type III secretion system and its direct interaction with the EspA-sheath-like structure. *Proc Natl Acad Sci* 98:11638–11643.
